# Research on Multi-Sensor Fusion Localization for Forklift AGV Based on Adaptive Weight Extended Kalman Filter

**DOI:** 10.3390/s25185670

**Published:** 2025-09-11

**Authors:** Qiang Wang, Junqi Wu, Yinghua Liao, Bo Huang, Hang Li, Jiajun Zhou

**Affiliations:** School of Mechanical Engineering, Sichuan University of Science and Engineering, Zigong 643000, China; wangqiangsuse@suse.edu.cn (Q.W.); wujunqi339330@163.com (J.W.); huangbojx@suse.edu.cn (B.H.); lihang@suse.edu.cn (H.L.); z2438782874@163.com (J.Z.)

**Keywords:** automated guided vehicles, multi-sensor fusion, Extended Kalman Filter, adaptive localization

## Abstract

This study addresses the problem localization deviation caused by cumulative wheel odometry errors in Automated Guided Vehicles (AGVs) operating in complex environments by proposing an adaptive localization method based on multi-sensor fusion. Within an Extended Kalman Filter (EKF) framework, the proposed approach integrates internal sensor predictions with external positioning data corrections, employing an adaptive weighting algorithm to dynamically adjust the contributions of different sensors. This effectively suppresses errors induced by factors such as ground friction and uneven terrain. The experimental results demonstrate that the method achieves a localization accuracy of 13 mm, and the simulation results show a higher accuracy of 10 mm under idealized conditions. The minor discrepancy is attributed to unmodeled noise and systematic errors in the complex real-world environment, thus validating the robustness of the proposed approach while maintaining robustness against challenges such as Non-Line-of-Sight (NLOS) obstructions and low-light conditions. The synergistic combination of LiDAR and odometry not only ensures data accuracy but also enhances system stability, providing a reliable navigation solution for AGVs in industrial settings.

## 1. Introduction

With the rapid development of industrial automation, Automated Guided Vehicles (AGVs) have become a core component of modern logistics and warehousing systems [1,2,3]. Indoor forklift-type AGVs particularly rely on high-precision positioning technology to ensure safe and efficient material handling [4,5,6]. However, complex and dynamic indoor environments (e.g., dynamic obstacles, reflective surfaces, and lighting variations) pose significant challenges to robust positioning for single-sensor systems [7,8,9]. Since individual sensors inevitably exhibit systematic or measurement errors, multi-sensor fusion technology has emerged as a research hotspot due to its complementary advantages [10,11,12].

This study presents a novel Kalman-filter-based multi-sensor data fusion framework that effectively combines measurements from heterogeneous sensors with either identical or distinct measurement matrices, thereby significantly improving state estimation accuracy while reducing noise interference in target tracking applications [13]. One study presented a tightly coupled fusion algorithm for Ultra Wide Band (UWB) and LiDAR Simultaneous Localization and Mapping (SLAM) incorporating NLOS identification. In contrast to such methods that rely on UWB infrastructure, our proposed approach is designed for environments where pre-deployed UWB base stations are not available, focusing instead on a fusion between LiDAR and odometry [14,15]. This study proposes a loosely coupled integration method using an Extended Kalman Filter (EKF) to fuse Inertial Navigation System (INS) and LiDAR SLAM, significantly reducing positioning errors in GNSS-challenged environments such as urban and highway scenarios [16].

Regarding weight adjustment: Methods like Kalman Filter Long Short-term Memory (KF-LSTM) rely on pre-trained, static neural networks for implicit error correction, lacking a mechanism for real-time adaptation to changing sensor reliability [17,18]. Multilevel-SLAM and similar optimization-based approaches depend on fixed covariance parameters tuned offline [19]. Conversely, our AWEKF algorithm introduces an explicit, lightweight, and dynamic weight adjustment mechanism. It calculates a confidence factor in real-time based on the innovation sequence, autonomously adjusting the influence of each sensor measurement at every filter iteration to suppress outliers and handle intermittent sensor degradation.

Regarding sensor compatibility and dependency: Many systems, such as KF-LSTM and tightly coupled UWB/LiDAR [20,21], are heavily dependent on specific external infrastructure like UWB anchor networks, rendering them inoperative if that infrastructure is unavailable or malfunctioning. Other methods are specialized for particular sensor pairs (e.g., LiDAR Inertial Measurement Unit (IMU) [22]). In comparison, the AWEKF framework is inherently more flexible and self-contained. It is primarily designed for the common and practical sensor suite of LiDAR and odometry, which are standard onboard sensors for most AGVs, eliminating the need for pre-installed external hardware.

Regarding computational efficiency, approaches leveraging deep learning (LSTM) or large-scale graph optimization can incur significant computational overhead, challenging their deployment on embedded systems for real-time AGV control. Our solution, by contrast, builds upon the computationally efficient EKF framework. The added adaptive component introduces minimal overhead, ensuring high-frequency operation and making it highly suitable for real-time industrial applications.

This study addresses the positioning challenges of indoor fork-lift AGVs by proposing a multi-sensor fusion localization method based on an Adaptive Weight Extended Kalman Filter (AWEKF). LiDAR provides accurate distance measurements and environmental feature in-formation but may exhibit positioning jumps in dynamic environments or during sensor failures. Odometry offers excellent short-term accuracy and continuity but suffers from cumulative errors. The approach fully leverages LiDAR’s high-precision environmental perception capabilities and odometry’s continuous motion estimation characteristics, achieving complementary advantages through sensor information fusion.

The proposed AWEKF algorithm dynamically adjusts fusion weights according to real-time sensor reliability, effectively handling time-varying and uncertain noise characteristics. Compared with traditional fixed-weight fusion methods, this algorithm demonstrates superior robustness and adaptability, maintaining stable positioning performance during partial sensor failures or environmental interference. It provides crucial technical support for precise positioning and safe operation of indoor forklift AGVs.

The remainder of this paper is organized as follows. Section 2 elaborates on the positioning principles of the odometer and the LiDAR system. Section 3 details the proposed AWEKF fusion localization algorithm, including the prediction model, measurement model, and the core adaptive strategy. Section 4 presents the simulation and experimental results alongside a comprehensive discussion. Finally, Section 5 concludes the paper and suggests directions for future work.

## 2. Positioning Principles of Sensors

### 2.1. Odometer Positioning

Based on differences in sensing principles, odometry systems can be systematically categorized into several types, primarily including wheel odometry, inertial odometry, laser odometry, and visual odometry. Among these, wheel odometry mechanically couples rotary encoders with the reduction gear of drive motors, utilizing wheel rotation to generate quadrature pulse signals from the encoders. Through analysis and processing of these pulse sequences, the control system can accurately derive kinematic parameters such as angular displacement and angular velocity of the drive wheels.

The wheel odometry system employed in this study utilizes an AB-phase quadrature incremental optical encoder. This sensing device captures real-time angular velocity and angular displacement information of the rotating shaft through photoelectric conversion principles. Following algorithmic processing by the controller, it can precisely calculate the actual displacement of the forklift-type AGV within specific time intervals. Assuming the wheel displacement per pulse of the encoder is γ (unit: mm/pulse), its mathematical model can be expressed as

(1) $$\gamma =\frac{2\pi R}{\eta E}$$

In Equation (1), *R* represents the outer radius of the steering wheel in the forklift-type AGV (unit: mm), *E* denotes the number of pulses generated per revolution of the incremental encoder (unit: pulse/rev), and η is defined as the reduction ratio of the motor’s gearbox. Based on these parameters, Equation (2) can accurately calculate the linear displacement Δs (unit: mm) of the steering wheel within a given time interval Δt, where Δn indicates the pulse count acquired during the corresponding time window (unit: pulse).

(2) Δs=Δnγ

**Figure 1:**
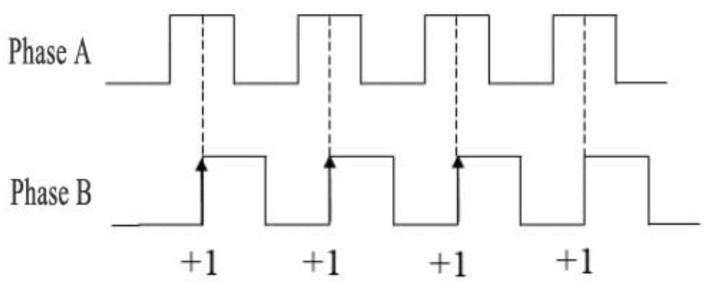
Principle diagram of encoder forward rotation with counting.

The incremental encoder employed in this system outputs three-channel digital signals: Phase A, Phase B quadrature pulse signals, and a Phase Z index signal. Specifically, Phase A and Phase B output square-wave pulse sequences with a 90° phase difference, while Phase Z generates a reference signal consisting of a single pulse per revolution. By detecting the phase-lead relationship between Phase A and Phase B signals, the motion controller can accurately determine the rotational direction of the driven wheel. Figure 1 presents the schematic diagram of the steering discrimination mechanism based on the AB-phase quadrature encoding principle.

As shown in Figure 1, the phase of Signal A leads that of Signal B by 90 degrees, indicating that the odometer is operating in forward rotation mode. When the motion detection system fully captures the square-wave signals from both Phase A and Phase B, the direction discrimination circuit outputs a forward-counting pulse.

**Figure 2:**
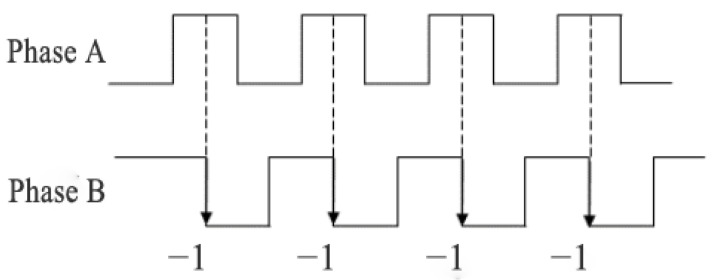
Principle diagram of encoder reverse rotation with counting.

As shown in Figure 2, when the phase of Signal A lags that of Signal B by 90 degrees, this indicates the odometer is operating in reverse rotation mode. At this point, when the motion detection system acquires the square-wave signals from both Phase A and Phase B, the direction discrimination circuit generates a reverse-counting pulse.

Based on the kinematic model of the forklift-type AGV, the main control system can accurately compute the motion state parameters of the drive wheels after receiving the real-time data stream from the wheel odometry. Through coordinate transformation and map-matching algorithms, the system determines its actual position relative to the initial pose within the pre-built environmental map.

However, under actual operating conditions, measurement errors inevitably accumulate in the wheel odometry data due to several factors: wheel slippage caused by uneven ground, inherent errors in the mechanical transmission system, and encoder quantization errors. Moreover, these errors exhibit time-divergent characteristics, leading to progressive degradation in positioning accuracy. To address this issue, the system employs LiDAR for periodic global pose correction, effectively reducing odometric error accumulation through multi-sensor data fusion.

### 2.2. LiDAR Positioning

**Figure 3:**
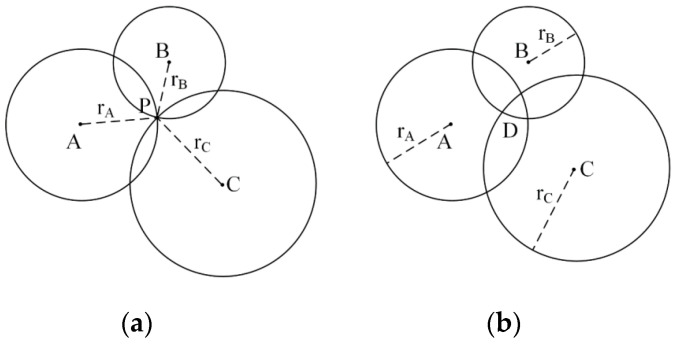
Principle of three-sided positioning: (**a**) Three circles intersecting at a single point; (**b**) three circles intersecting within a common area.

The mobile robot system employs a reflector-based trilateration method for global pose estimation. As illustrated in Figure 3a, let $A(x_{A},y_{A})$, $B(x_{B},y_{B})$, and $C(x_{C},y_{C})$ represent the planar coordinates of three reflector reference points acquired through LiDAR scanning, with $P(x_{P},y_{P})$ denoting the robot’s current position. The measured Euclidean distances from these reference points to the robot are $r_{A}$, $r_{B}$, and $r_{C}$, respectively. According to the trilateration principle, three positioning circles are constructed using each reference point’s coordinates as the center and the corresponding measured distance as the radius. The intersection point of these circles determines the coordinate solution for the robot’s current position P. The mathematical formulation of this positioning algorithm can be expressed as the following system of equations:

(3) $$\left\{\begin{array}{l} {\left(x_{P}-x_{A}\right)}^{2}+{\left(y_{P}-y_{A}\right)}^{2}=r_{A}^{2}\\ {\left(x_{P}-x_{B}\right)}^{2}+{\left(y_{P}-y_{B}\right)}^{2}=r_{B}^{2}\\ {\left(x_{P}-x_{C}\right)}^{2}+{\left(y_{P}-y_{C}\right)}^{2}=r_{C}^{2} \end{array}\right.$$

Under ideal conditions, the unique position $P(x_{P},y_{P})$ of the mobile robot can be determined by solving Equation (3), while its deflection angle *θ* can be calculated based on reflector *A*’s coordinates as follows:

(4) $$\theta_{A}=arc\tan\frac{x_{A}-x_{P}}{y_{A}-y_{P}}-\varphi_{A}$$

In the above equation, $\varphi_{A}$ represents the angular position of reflector *A* in the LiDAR polar coordinate system. Similarly, the angular positions $\theta_{B}$ and $\theta_{C}$ can be determined, yielding the mobile robot’s average deflection angle as follows:

(5) $$\theta_{P}=\frac{1}{3}\left(\theta_{A}+\theta_{B}+\theta_{C}\right)$$

In practical scenarios, measurement errors prevent the three circles from intersecting at a single point, resulting in either: (1) an overlapping region D (Figure 3b), (2) non-intersecting circles, or (3) detection of more than three reflectors by the LiDAR. Under these conditions, Equation (3) forms an overdetermined system with no exact solution, necessitating alternative solution algorithms. This paper employs the least squares method to compute an approximate solution, assuming the LiDAR detects i (i≥3) reflectors.

(6) $$\left\{\begin{array}{c} {\left(x_{P}-x_{1}\right)}^{2}-{\left(y_{P}-y_{1}\right)}^{2}=r_{1}^{2}\\ \vdots \\ {\left(x_{P}-x_{n}\right)}^{2}+{\left(y_{P}-y_{n}\right)}^{2}=r_{n}^{2} \end{array}\right.$$

Expand each equation:

(7) $$x_{P}^{2}-2x_{i}x_{P}+x_{i}^{2}+y_{P}^{2}-2y_{i}y_{P}+y_{i}^{2}=r_{i}^{2}$$

Rearranged as:

(8) $$\begin{array}{cc} x_{P}^{2}+y_{p}^{2}-2x_{i}x_{P}-2y_{i}y_{P}=r_{i}^{2}-x_{i}^{2}-y_{i}^{2} & \left(i=1,2,\cdots ,n\right) \end{array}$$

Eliminate the quadratic term $x_{P}^{2}+y_{P}^{2}$ by subtracting the *i*th equation minus the nth equation:

(9) $$\begin{array}{c} \left(x_{P}^{2}+y_{P}^{2}-2x_{i}x_{P}-2y_{i}y_{P})-(x_{P}^{2}+y_{P}^{2}-2x_{n}x_{P}-2y_{n}y_{P}\right)\\ =(r_{i}^{2}-x_{i}^{2}-y_{i}^{2})-(r_{n}^{2}-x_{n}^{2}-y_{n}^{2}) \end{array}$$

After simplification:

(10) $$-2x_{i}x_{P}-2y_{i}y_{P}+2x_{n}x_{P}+2y_{n}y_{P}=r_{i}^{2}-x_{i}^{2}-y_{i}^{2}-r_{n}^{2}+x_{n}^{2}+y_{n}^{2}$$

Further tidying up:

(11) $$2(x_{i}-x_{n})x_{P}+2(y_{i}-y_{n})y_{P}=x_{i}^{2}-x_{n}^{2}+y_{i}^{2}-y_{n}^{2}+r_{n}^{2}-r_{i}^{2}$$

For *i* = 1, 2, …, (n − 1), we obtain the linear system of equations:

(12) $$\left(\begin{array}{c} 2(x_{1}-x_{n})x_{P}+2(y_{1}-y_{n})y_{P}=x_{1}^{2}-x_{n}^{2}+y_{1}^{2}-y_{n}^{2}+r_{n}^{2}-r_{1}^{2}\\ \vdots \\ 2(x_{n-1}-x_{n})x_{P}+2(y_{n-1}-y_{n})y_{P}=x_{n-1}^{2}-x_{n}^{2}+y_{n-1}^{2}-y_{n}^{2}+r_{n}^{2}-r_{n-1}^{2} \end{array}\right.$$

We derive the following linearized system by subtracting the nth equation from the first n−1 equations:

(13) $$AX_{P}=b$$

where

(14) $$A=\left[\begin{array}{cc} 2(x_{1}-x_{n}) & 2(y_{1}-y_{n})\\ \vdots & \vdots \\ 2(x_{n-1}-x_{n}) & 2(y_{n-1}-y_{n}) \end{array}\right]$$

(15) $$b=\left[\begin{array}{c} x_{1}^{2}-x_{n}^{2}+y_{1}^{2}-y_{n}^{2}+r_{n}^{2}-r_{1}^{2}\\ \vdots \\ x_{n-1}^{2}-x_{n}^{2}+y_{n-1}^{2}-y_{n}^{2}+r_{n}^{2}-r_{n-1}^{2} \end{array}\right]$$

The least squares method can subsequently be employed to solve the linear system represented by Equation (7):

(16) $$A^{T}AX_{P}=A^{T}b$$

Least squares solution:

(17) $$X_{p}={\left(A^{T}A\right)}^{-1}A^{T}b$$

Similarly, the mobile robot’s deflection angle $\theta_{P}$ can be computed as follows:

(18) $$\theta_{p}=\frac{1}{n}\sum_{i=1}^{n}arc\tan\frac{x_{i}-x_{P}}{y_{i}-y_{P}}-\varphi_{i}$$

## 3. Improved EKF Fusion Localization

**Figure 4:**
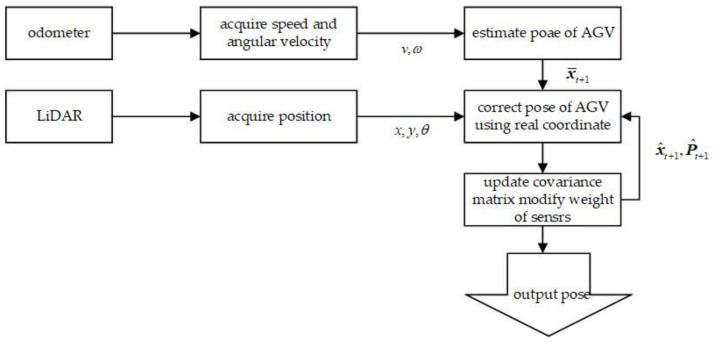
General framework of EKF fusion localization system.

Wheel odometry inevitably accumulates errors during inertial navigation, with the magnitude of these errors increasing proportionally with travel distance. Consequently, this method cannot satisfy the accuracy requirements for mobile robots performing long-range positioning tasks. Furthermore, reflector-based LiDAR global localization systems exhibit three inherent limitations: (1) limited update frequency (typically 50 Hz); (2) significant pose estimation discrepancies; and (3) degraded localization performance when reflectors are either occluded or displaced. To overcome these challenges, this study implements a multi-sensor fusion framework based on the Extended Kalman Filter (EKF), as shown in Figure 4, in which wheel odometry provides linear v and angular velocity ω measurements while LiDAR enables global localization to obtain absolute pose information $(\begin{array}{ccc} x & y & \theta ) \end{array}$. The proposed algorithm effectively compensates for the nonlinear dynamics inherent in mobile robot systems, achieving both substantial improvements in positioning accuracy and robustness while maintaining real-time computational performance.

Initialize the sensor model and parameters:

### 3.1. Odometer Prediction Model

During AGV operation, the system state can be formally represented by the following state-space vector:

(19) $$x_{t}={\left(\begin{array}{ccc} x_{t} & y_{t} & \theta_{t} \end{array}\right)}^{T}$$

In the world coordinate frame, the state vector of the automated guided vehicle (AGV) at time t is represented as $x_{t}$, where $x_{t}$ denotes the position on the x-axis, $y_{t}$ denotes the position on the y-axis, and $\theta_{t}$ is the heading angle. Under the condition of linear motion, where the angular velocity ω=0, the state transition model derived from odometry is defined as follows:

(20) $$x_{t+1}=f(x_{t},u_{t+1})=\left(\begin{array}{c} x_{t}\\ y_{t}\\ \theta_{t} \end{array}\right)+\left(\begin{array}{c} \nu_{t+1}\Delta t\cos\theta_{t}\\ \nu_{t+1}\Delta t\sin\theta_{t}\\ 0 \end{array}\right)$$

The state vector $x_{t+1}=f(x_{t},u_{t+1})$ denotes the pose of the automated guided vehicle (AGV) at time step t+1. This state is predicted using the motion control input $u_{t+1}={\left(\begin{array}{cc} v_{t+1} & \omega_{t+1} \end{array}\right)}^{T}$, where $v_{t+1}$ and $\omega_{t+1}$ represent the linear and angular velocity, respectively.

A linear approximation of the transition function is applied at the AGV’s posterior pose estimate from the previous time step, which yields the corresponding Jacobian matrix:

(21) $$F_{t+1}=\frac{\partial f}{\partial x_{t}}{|}_{{\hat{x}}_{t}}=\left(\begin{array}{ccc} 1 & 0 & -\nu_{t+1}\Delta t\sin({\hat{\theta }}_{t})\\ 0 & 1 & \nu_{t+1}\Delta t\cos({\hat{\theta }}_{t})\\ 0 & 0 & 1 \end{array}\right)$$

$F_{t+1}$ is the Jacobian matrix of the nonlinear state transition function with respect to the state vector ${\hat{x}}_{t}$. It is evaluated using the current control input $u_{t+1}$ and the state estimate $x_{t}$.

The formal definition of the odometric noise covariance matrix, $M_{t+1}$, is provided below.

(22) $$M_{t+1}=\left[\begin{array}{cc} a_{1}v_{t+1}^{2} & 0\\ 0 & a_{3}v_{t+1}^{2} \end{array}\right]$$

Parameters $a_{1}$ and $a_{3}$ are robot-specific motion error coefficients. Their values should be selected based on the specific kinematic properties of the automated guided vehicle (AGV).

By linearizing the function $f({\hat{x}}_{t},u_{t+1})$ with respect to the motion parameters $v_{t+1}$ and $\omega_{t+1}$, we obtain the Jacobian matrix $V_{t+1}$ as follows:

(23) $$V_{t+1}=\frac{\partial f}{\partial (\nu ,\omega )}{|}_{\left(\nu_{t+1},\omega_{t+1}\right)}=\left[\begin{array}{cc} \Delta t\cos({\hat{\theta }}_{t}) & 0\\ \Delta t\sin({\hat{\theta }}_{t}) & 0\\ 0 & 0 \end{array}\right]$$

Consequently, the predicted covariance matrix resulting from odometric motion noise $Q_{t+1}$ can be computed as follows:

(24) $$Q_{t+1}=V_{t+1}M_{t+1}V_{t+1}^{T}$$

When ω≠0, the AGV’s state transition function can be expressed as

(25) $$\left.f(x_{t},u_{t+1})=\left(\begin{array}{c} x_{t}\\ y_{t}\\ \theta_{t} \end{array}\right.\right)+\left(\begin{array}{c} -\frac{\nu_{t+1}}{\omega_{t+1}}\sin\theta_{t}+\frac{\nu_{t+1}}{\omega_{t+1}}\sin(\theta_{t}+\omega_{t+1}\Delta t)\\ \frac{\nu_{t+1}}{\omega_{t+1}}\cos\theta_{t}-\frac{\nu_{t+1}}{\omega_{t+1}}\cos(\theta_{t}+\omega_{t+1}\Delta t)\\ \omega_{t+1}\Delta t \end{array}\right)$$

In this study, the AGV’s motion is controlled by adjusting the input vector $u_{t+1}={\left(\nu_{t+1}\;\omega_{t+1}\right)}^{T}$. Through Taylor expansion linearization of the nonlinear state prediction model for the forklift-type AGV within the Extended Kalman Filter (EKF) localization framework, we derive the following formulation:

(26) $$f(x_{t},u_{t+1})\approx f({\hat{x}}_{t},u_{t+1})+F_{t+1}(x_{t}-{\hat{x}}_{t})$$

In this formulation, the function $f(x_{t},u_{t+1})$ approximates the unknown true state $x_{t}$ using the known expected control input ${\hat{x}}_{t}$. The Jacobian matrix $F_{t+1}$ represents the partial derivative of the nonlinear function with respect to state ${\hat{x}}_{t}$, evaluated at the current control input $u_{t+1}$ and state estimate $x_{t}$:

(27) $${\left.F_{t+1}=\frac{\partial f}{\partial x_{t}}\right|}_{{\hat{x}}_{t}}=\left(\begin{array}{ccc} 1 & 0 & \frac{\nu_{t+1}}{\omega_{t+1}}(-\cos{\hat{\theta }}_{t}+\cos({\hat{\theta }}_{t}+\omega_{t+1}\Delta t))\\ 0 & 1 & \frac{\nu_{t+1}}{\omega_{t+1}}(-\sin{\hat{\theta }}_{t}+\sin({\hat{\theta }}_{t}+\omega_{t+1}\Delta t))\\ 0 & 0 & 1 \end{array}\right)$$

The odometer noise covariance matrix $M_{t+1}$ associated with the control input is formally defined as follows:

(28) $$M_{t+1}=\left[\begin{array}{cc} a_{1}\nu_{t+1}^{2}+a_{2}\omega_{t+1}^{2} & 0\\ 0 & a_{3}\nu_{t+1}^{2}+a_{4}\omega_{t+1}^{2} \end{array}\right]$$

Parameters $a_{1}$, $a_{2}$, $a_{3}$, and $a_{4}$ are robot-specific motion error coefficients. Their values should be calibrated based on the specific kinematic properties of the automated guided vehicle (AGV).

By linearizing the nonlinear function $f({\hat{x}}_{t},u_{t+1})$ with respect to the motion parameters $v_{t+1}$ (linear velocity) and $\omega_{t+1}$ (angular velocity), we derive the Jacobian matrix $V_{t+1}$ as follows:

(29) $$\begin{array}{ll} V_{t+1} & =\frac{\partial f}{\partial (\nu ,\omega )}{|}_{\left(\nu_{t+1},\omega_{t+1}\right)}\\ & =\left[\begin{array}{ll} \frac{\partial f_{x}}{\partial v_{t+1}} & \frac{\partial f_{x}}{\partial \omega_{t+1}}\\ \frac{\partial f_{y}}{\partial v_{t+1}} & \frac{\partial f_{y}}{\partial \omega_{t+1}}\\ \frac{\partial f_{\theta }}{\partial v_{t+1}} & \frac{\partial f_{\theta }}{\partial \omega_{t+1}} \end{array}\right]\\ & =\left[\begin{array}{cc} \frac{-\sin{\hat{\theta }}_{t}+\sin({\hat{\theta }}_{t}+\omega_{t+1}\Delta t)}{\omega_{t+1}} & \frac{\nu_{t+1}(\sin{\hat{\theta }}_{t}-\sin({\hat{\theta }}_{t}+\omega_{t+1}\Delta t)}{\omega_{t+1}^{2}}+\frac{\nu_{t+1}\cos({\hat{\theta }}_{t}+\omega_{t+1}\Delta t)\Delta t}{\omega_{t+1}}\\ \frac{\cos{\hat{\theta }}_{t}-\cos({\hat{\theta }}_{t}+\omega_{t+1}\Delta t)}{\omega_{t+1}} & -\frac{\nu_{t+1}(\cos{\hat{\theta }}_{t}-\cos({\hat{\theta }}_{t}+\omega_{t+1}\Delta t)}{\omega_{t+1}^{2}}+\frac{\nu_{t+1}\sin({\hat{\theta }}_{t}+\omega_{t+1}\Delta t)\Delta t}{\omega_{t+1}}\\ 0 & \Delta t \end{array}\right] \end{array}$$

Consequently, the prediction step in the EKF framework generates the following results:

(30) $${\bar{x}}_{t+1}=f({\hat{x}}_{t},u_{t+1})$$

(31) $${\bar{P}}_{t+1}=F_{t+1}{\hat{P}}_{t}F_{t+1}^{T}+Q_{t+1}$$

where ${\bar{x}}_{t+1}$ represents the state variable at time t+1, ${\bar{P}}_{t+1}$ is the covariance matrix of state estimation, $F_{t+1}$ is the system state transition matrix, and $Q_{t+1}$ is the process noise generated in the real situation, that is, the uncertainty of the outside world.

### 3.2. Lidar Measurement Model

Within the predefined global coordinate system of the factory environment, the proposed system utilizes NAV350 LiDAR sensors to continuously monitor the pose states of forklift-type AGVs at a constant sampling frequency. The sensor outputs pose observations ${\left(x\;y\;\theta \right)}^{T}$ at each measurement cycle. Let σ denote the standard deviation of the LiDAR’s ranging measurements, and $v_{t}$ represent a Gaussian white noise process. The LiDAR measurement model can be formally expressed as:

(32) $$z_{t}=h(x_{t})+v_{t}$$

Let H denote the Jacobian matrix of the nonlinear observation model, which can be expressed as:

(33) $$H=\left[\begin{array}{ccc} 1 & 0 & 0\\ 0 & 1 & 0\\ 0 & 0 & 1 \end{array}\right]$$

The measurement noise covariance matrix $R_{t}$ can be formally expressed as follows:

(34) $$R_{t}=\left[\begin{array}{ccc} \sigma^{2}/2 & 0 & 0\\ 0 & \sigma^{2}/2 & 0\\ 0 & 0 & \sigma_{\theta }^{2} \end{array}\right]$$

Since parameter $\sigma_{\theta }$ cannot be directly measured by the NAV350 LiDAR system, we select parameter σθ=σ3 as a suitable alternative. The state update procedure is therefore implemented through the following equations:

(35) $$K_{t+1}=P_{t+1}H_{t+1}^{T}{\left(H_{t+1}P_{t+1}H_{t+1}^{T}+R_{t+1}\right)}^{-1}$$

(36) $${\hat{x}}_{t+1}={\bar{x}}_{t+1}+K_{t+1}(z_{t+1}-h({\bar{x}}_{t+1}))$$

(37) $${\hat{P}}_{t+1}=(I-K_{t+1}H){\bar{P}}_{t+1}$$

where $K_{t+1}$ is called the Kalman gain, ${\hat{x}}_{t+1}$ is called the updated pose state, and $P_{t+1}$ is the covariance matrix of the state update.

### 3.3. Detect Sensor State and Modify Weight

The wheel odometer serves as an internal sensor, offering stable operation and high reliability. In contrast, external sensors may occasionally fail to provide positioning data. After each filtering iteration, the system dynamically adjusts the sensor weights based on their current operational status. When the LiDAR sensor provides valid positioning coordinates, the system utilizes these values to correct the pose estimate and update the covariance matrix. If the LiDAR sensor cannot provide positioning coordinates, its weight is set to zero, and the system propagates the pose based on the previous state. Once the AGV returns to the line-of-sight range and the LiDAR resumes providing positioning data, the system increases the LiDAR’s weight, updates the parameters of the LiDAR positioning covariance matrix using the value of $R_{t}$, and proceeds to the next round of prediction and positioning.

## 4. Simulation and Experimental Studies

### 4.1. Simulation Studies

This study developed a numerical simulation environment on the MATLAB R2018b platform, implementing the algorithmic architecture in M-language and visualizing the results through dedicated modules. Adopting a comparative methodology, we systematically evaluated performance differences among three localization approaches: (1) LiDAR-only localization, (2) odometry-only localization, and (3) EKF-based multi-sensor fusion. The simulation parameters were configured with 1 mm spatial resolution and 1 ms temporal resolution. The initial pose was defined by state vector $x={\left(\begin{array}{ccc} 0 & 0 & 0 \end{array}\right)}^{T}$, while motion control employed constant linear velocity 400 mm/s with a segmented angular velocity strategy (initial $\omega_{1}=0\;\mathrm{rad}/s$, adjusted $\omega_{2}=5\;\mathrm{rad}/s$), the system models incorporate zero-mean Gaussian white noise for both measurement and process uncertainties: LiDAR measurement noise is characterized by a standard deviation of $\sigma_{Lidar}=10\;\mathrm{mm}$ in position (x, y) and $\sigma_{\theta }=0.5\;\mathrm{rad}$ in heading, while odometry process noise is modeled with $\sigma_{v}=5\;\mathrm{mm}/s$ in linear velocity and $\sigma_{\omega }=0.3\;\mathrm{rad}/s$ in angular velocity. The initial state was set to ${\left[\begin{array}{ccc} 0 & 0 & 0 \end{array}\right]}^{T}$. The initial covariance P_0_ was set to diag$(\begin{array}{ccc} 1000 & 1000 & 0.5) \end{array}$ to represent large initial uncertainty. The experimental procedure involved four stages: (1) generating reference trajectories using ideal odometry as ground truth, (2) injecting zero-mean Gaussian white noise into LiDAR measurements to simulate real-world errors, (3) applying similar noise processing to odometry data, and (4) executing the EKF algorithm for optimal multi-sensor data fusion.

**Figure 5:**
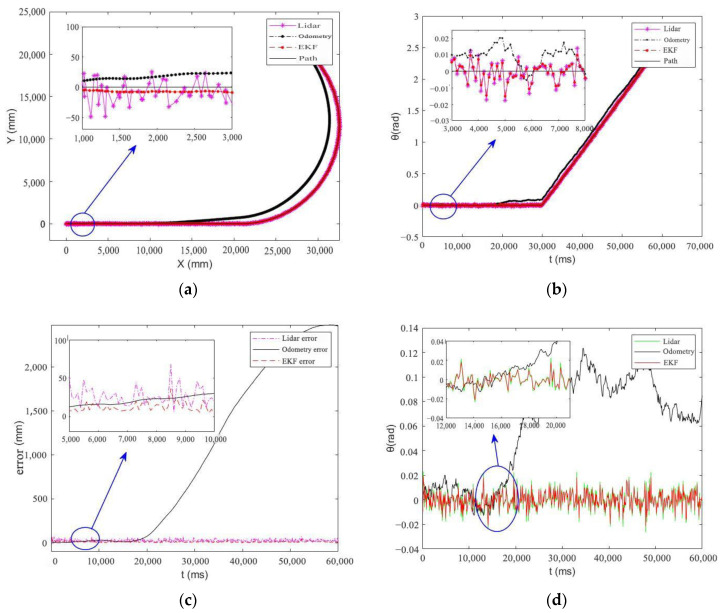
(**a**) Positioning trajectory; (**b**) heading angle; (**c**) trajectory error; (**d**) heading angle error.

Figure 5 presents the comparative trajectory results from Simulation Experiment a, demonstrates the temporal evolution of the positioning heading angle during AGV navigation in Simulation Experiment b, provides the positioning trajectory error analysis—including both absolute error and its estimated distribution—in Simulation Experiment c, and illustrates the heading angle error analysis by comparing measured values with ground truth references in Simulation Experiment d. These results collectively offer a comprehensive evaluation of the system’s navigation performance.

Analysis of the simulation results is presented in Table 1:

Systematic analysis of the simulation results (Figure 5 and Table 1) reveals three key findings: (1) In single-sensor configurations, the LiDAR-based localization achieves 28 mm positioning accuracy; (2) While the odometry system demonstrates higher short-term precision, its positioning error exhibits monotonic growth with travel distance; (3) The EKF-based multi-sensor fusion approach significantly improves system performance, attaining 10 mm positioning accuracy 64.3% enhancement compared to single-sensor solutions.

### 4.2. Experimental Studies

#### 4.2.1. Environment and Equipment

**Figure 6:**
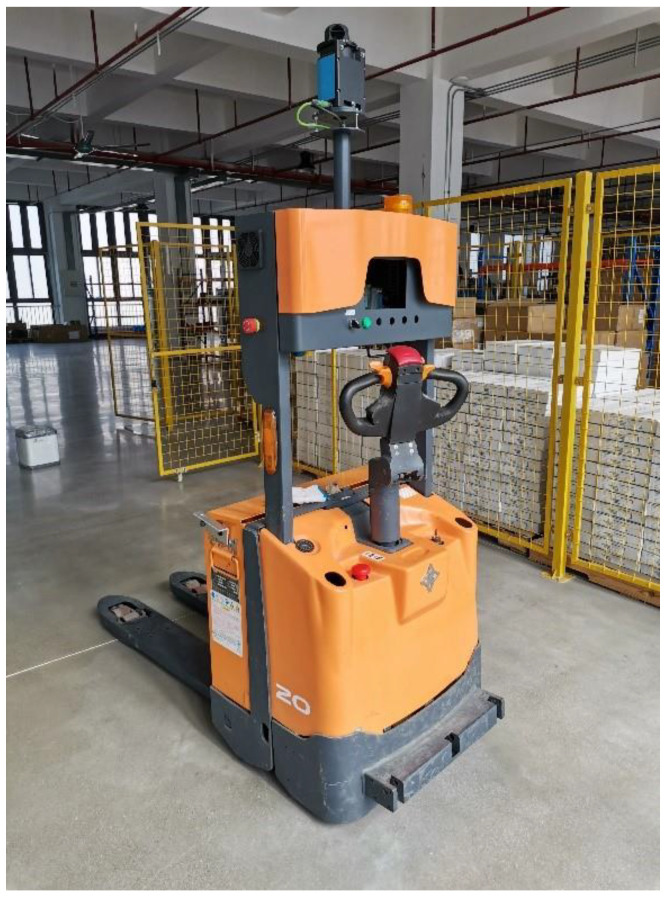
Physical map of forklift AGV.

The forklift-type AGV developed in this study integrates a NAV350 LiDAR navigation system—manufactured by SICK AG (Waldkirch, Germany)—for high-precision positioning at an update rate of 8 Hz. The drive system features a three-wheel front-drive configuration, with the active steering mechanism jointly controlled by an independent straight-line drive motor and a steering servo motor, enabling precise regulation of both linear velocity v and steering angle θ. During operation, incremental encoders mounted on the motor shafts acquire real-time velocity vectors and steering angles, transmitting these data to the PLC master controller via CAN bus communication. The PLC operates with a task cycle time of 10 ms. The experimental platform utilizes a modified Zowell industrial forklift chassis, equipped with our proprietary positioning/navigation and motion control systems, which has successfully demonstrated fully automated material handling in intelligent warehouse applications. Figure 6 illustrates the physical implementation of this system.

**Figure 7:**
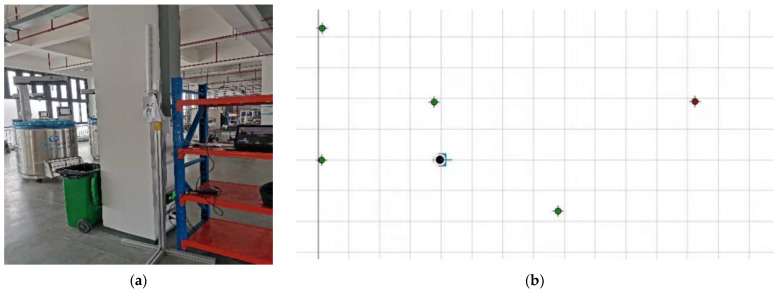
(**a**) Physical map of reflector; (**b**) map of Sopas ET.

The NAV350 LiDAR system implemented in this research utilizes cylindrical reflectors for global pose estimation. In the experimental setup phase, an array of reflectors was strategically deployed throughout the industrial environment following a predefined topological configuration, as illustrated in Figure 7. The map construction procedure consists of five sequential steps: (1) establishing an Ethernet connection between the NAV350 sensor and the host computer, (2) launching the professional mapping software Sopas ET 2.38, (3) entering debugging mode by inputting the device IP address, (4) configuring scanning parameters including detection radius (50 m) and reflector count (5 units), and (5) defining the origin of the global coordinate system to finalize the environmental map. The constructed map data is stored in the LiDAR’s onboard memory. Figure 7 presents the environmental map generated by the Sopas ET 2.38 software.

#### 4.2.2. Ground Truth Acquisition and Spatiotemporal Synchronization

1. Ground Truth Acquisition and Calibration

The experimental environment was initially mapped with high metric precision using a LiDAR sensor to construct a detailed reference map. During evaluation runs, the AGV utilized the same LiDAR-based internal algorithm to localize itself within this pre-built map, with the continuously optimized pose serving as the ground truth. Prior to data collection, extrinsic calibration was performed to accurately align the coordinate frames of the LiDAR and wheel odometry sensors, ensuring consistency in data fusion.

2. Spatiotemporal Synchronization

All sensors, including LiDAR and odometry, as well as the internal algorithm, were integrated under the Operating System TwinCat3. Operating System facilitated software-based synchronization by timestamping all sensor messages using the host computer’s clock. Although not hardware-level synchronization, this approach achieved millisecond-level temporal alignment, which is sufficient given the indoor AGV’s motion dynamics. The resulting synchronization error was negligible compared to the positioning errors under investigation.

#### 4.2.3. Fusion Localization Results

**Figure 8:**
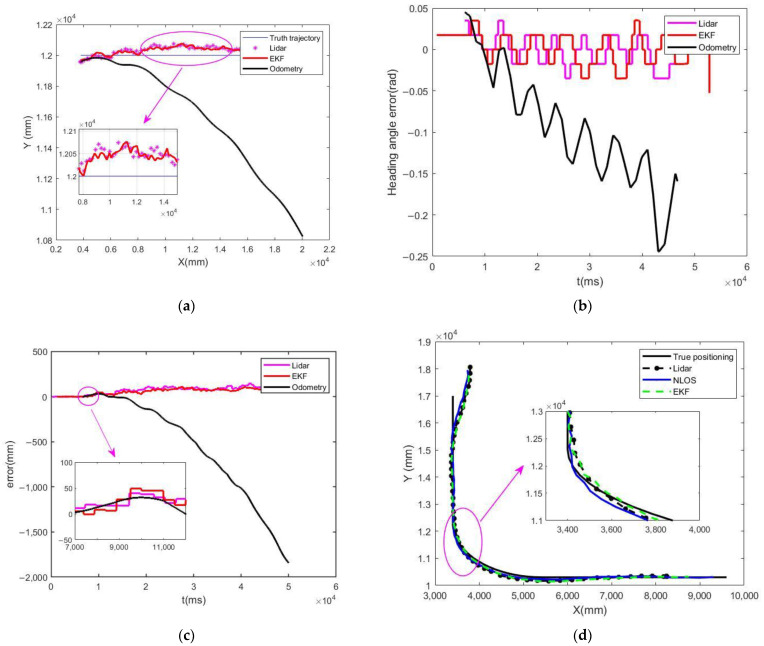
(**a**) Linear positioning position; (**b**) heading angle error; (**c**) positioning error; (**d**) turn position positioning.

To quantitatively assess the localization performance of the Extended Kalman Filter (EKF) multi-sensor fusion algorithm, we conducted comparative experiments evaluating three distinct approaches: (1) wheel odometry-based standalone localization, (2) LiDAR-based standalone localization, and (3) EKF-fused LiDAR-odometry localization. The experimental parameters were configured with the forklift-type AGV maintaining a constant linear velocity of 400 mm/s and zero steering angle (0°) for straight-line trajectory tracking. Motion capture system (accuracy: ±0.1 mm) served as the ground truth reference for pose data acquisition. Figure 8a,d present the positional accuracy comparison during straight-line motion and turning motion, while Figure 8b,c, respectively, illustrate the temporal evolution of heading angle estimation errors and positional deviations across the three localization schemes.

A comparative analysis of Figure 8 reveals that under short-distance motion conditions, the odometry-only localization system demonstrates relatively high positioning accuracy. However, as the travel distance increases, the positioning accuracy of odometry degrades significantly. In contrast, while LiDAR standalone localization similarly achieves high initial precision, it suffers from data update latency between scanning cycles and is susceptible to interference from reflector noise. By incorporating the Extended Kalman Filter (EKF) algorithm to fuse LiDAR and odometry data, this study achieves real-time interpolation of NAV350 positioning results. This approach not only significantly improves positioning frequency but also optimizes localization accuracy, effectively mitigating the adverse effects of reflector noise.

To quantify the positioning accuracy of the forklift-type AGV, this study systematically collected localization error data during operation and performed an error distribution analysis. Under identical experimental conditions, multi-sensor error data after EKF fusion and pure odometry localization errors were recorded. The detailed statistical error analysis results are presented in Table 2.

Based on the positioning accuracy analysis results presented in Table 2, during dynamic localization, the LiDAR system—subject to environmental noise interference—could only maintain positioning accuracy within a range of 21 mm. However, after implementing the Extended Kalman Filter (EKF) algorithm to optimize the fusion of LiDAR and odometry data, the positioning accuracy was significantly improved to 13 mm, thereby substantially enhancing the AGV’s localization performance.

A comparative analysis of the results presented in Table 1 and Table 2 reveals a minor performance discrepancy between the simulation (10 mm mean error) and experimental (13 mm mean error) environments. This 3 mm deviation is attributed to the inherent idealization of the simulation model versus the complexities of the real-world setting. The simulation operated under several optimal assumptions: a perfectly flat ground surface eliminating wheel slip, Gaussian white noise models for both odometry and LiDAR measurements, and flawless detection and identification of all reflectors. Conversely, the physical experiment introduced unmodeled challenges: slight ground irregularities inducing wheel slippage and odometry error, systematic errors from sensor misalignment and imperfect calibration, and real LiDAR point cloud artifacts such as occasional misidentification of reflectors or spurious returns from reflective surfaces. The fact that the AWEKF algorithm maintained a high level of accuracy (13 mm) in the face of these additional real-world perturbations, with only a minimal performance loss compared to the idealized simulation, strongly underscores its robustness and practical viability. The observed deviation does not diminish the result but rather validates the algorithm’s effectiveness in transitioning from a theoretical model to a practical application. The spikes in LiDAR error in Figure 8a,d are caused by transient reflectors occlusion. AWEKF algorithm’s ability to suppress these spikes and maintain stable performance demonstrates its capability to handle intermittent NLOS-like disturbances.

## 5. Conclusions

This study proposes an adaptive-weight Extended Kalman Filter (EKF)-based multi-sensor fusion localization framework. By integrating LiDAR and odometry data, it effectively mitigates pose estimation deviations in indoor forklift AGVs caused by odometric error accumulation, ground friction dynamics variations, and mechanical vibrations. The methodological innovation lies in its internal-prediction/external-correction fusion strategy and adaptive weight optimization algorithm, which dynamically adjusts fusion weights according to real-time sensor confidence levels, achieving complementary advantages of multi-source information. Experimental validation demonstrates that compared with single-sensor localization schemes, the proposed fusion algorithm significantly improves positioning accuracy, with measured positioning errors controlled within 13 mm and further optimized to 10 mm in simulation environments, fully meeting the high-precision pose estimation requirements for indoor forklift AGVs. Moreover, the system maintains robustness under complex operating conditions, including non-line-of-sight (NLOS) landmark occlusion and low-light environments: LiDAR ensures spatial accuracy of fused data while odometry preserves temporal stability of the system. This research provides a reliable engineering solution for precision localization and safe operation of indoor forklift AGVs.

## Figures and Tables

**Table 1:** Data comparison of positioning results.

Positioning Methods	Mean Error of Positioning Trajectory	Maximum Positioning Trajectory Error	Minimum Trajectory Positioning Error
Lidar	28 mm	57 mm	1.2 mm
Odometry	100 mm	2477 mm	0.9 mm
EKF	10 mm	29 mm	0.6 mm

**Table 2:** Analysis of Positioning Accuracy.

Positioning Methods	Mean Error of Positioning Trajectory	Maximum Positioning Trajectory Error	Minimum Trajectory Positioning Error
Lidar	21 mm	70 mm	1 mm
Odometry	921 mm	1843 mm	2 mm
EKF	13 mm	61 mm	1 mm

## Data Availability

Data are contained within the article.
